# Supplementary material for: Iterative improvement in the automatic modular design of robot swarms
Source: PeerJ Comput Sci. 2020 Dec 7;6:e322. doi: 10.7717/peerj-cs.322 (PMC7924708; doi:10.7717/peerj-cs.322)
Supplement: Supplemental Information 3 [file peerj-cs-06-322-s003.zip › argos3/doc/api/standalone/a00443_source.html]

ARGoS: plugins/robots/foot-bot/control\_interface/ci\_footbot\_gripper\_actuator.h Source File


- Main Page
- Related Pages
- Namespaces
- Classes
- Files

- File List
- File Members

# plugins/robots/foot-bot/control\_interface/ci\_footbot\_gripper\_actuator.h

Go to the documentation of this file.

```
00001 
00015 #ifndef CCI_FOOTBOT_GRIPPER_ACTUATOR_H
00016 #define CCI_FOOTBOT_GRIPPER_ACTUATOR_H
00017 
00018 /* To avoid dependency problems when including */
00019 namespace argos {
00020    class CCI_FootBotGripperActuator;
00021 }
00022 
00023 #include <argos3/core/control_interface/ci_actuator.h>
00024 #include <argos3/core/utility/math/angles.h>
00025 
00026 namespace argos {
00027 
00028    class CCI_FootBotGripperActuator : virtual public CCI_Actuator {
00029 
00030    public:
00031 
00032       /* Useful gripper aperture values */
00033       static CRadians UNLOCKED;
00034       static CRadians LOCKED_POSITIVE;
00035       static CRadians LOCKED_NEGATIVE;
00036 
00037    public:
00038 
00042       CCI_FootBotGripperActuator();
00043 
00047       virtual ~CCI_FootBotGripperActuator() {}
00048 
00053       void SetAperture(const CRadians& c_aperture);
00054 
00058       virtual void EnableCheckForObjectGrippedRoutine() = 0;
00059 
00063       virtual void DisableCheckForObjectGrippedRoutine() = 0;
00064 
00068       void LockPositive();
00069 
00073       void LockNegative();
00074 
00078       void Unlock();
00079 
00080 #ifdef ARGOS_WITH_LUA
00081       virtual void CreateLuaState(lua_State* pt_lua_state);
00082 #endif
00083 
00084    protected:
00085 
00086       CRadians m_cAperture;
00087 
00088    };
00089 
00090 }
00091 
00092 #endif
```

---

Generated on 10 Jul 2018 for ARGoS by 
 1.6.1
